# LncRNA RUSC1-AS1 promotes osteosarcoma progression through regulating the miR-340-5p and PI3K/AKT pathway

**DOI:** 10.18632/aging.203047

**Published:** 2021-05-28

**Authors:** Chang-Jun Tong, Qing-Chun Deng, Di-Jun Ou, Xia Long, He Liu, Kang Huang

**Affiliations:** 1 Department of Orthopedics, Huazhong University of Science and Technology Union Shenzhen Hospital, Shenzhen 518000, China; 2 Department of Gynecology, The Second Affiliated Hospital of Hainan Medical University, Haikou 570102, China; 3 Department of Operating Room, Huazhong University of Science and Technology Union Shenzhen Hospital, Shenzhen 518000, China

**Keywords:** RUSC1-AS1, miR-340-5p, PI3K/AKT, osteosarcoma

## Abstract

Dysregulation of long noncoding RNA (lncRNA) is frequently involved in the progression and development of osteosarcoma. LncRNA RUSC1-AS1 is reported to be upregulated and acts as an oncogene in hepatocellular carcinoma, cervical cancer and breast cancer. However, its role in osteosarcoma has not been studied yet. In the present study, we investigated the role of RUSC1-AS1 in osteosarcoma both *in vitro* and *in vivo*. The results showed that the expression of RUSC1-AS1 was significantly upregulated in osteosarcoma cell line U2OS and HOS compared to that in human osteoblast cell line hFOB1.19. Similar results were found in human samples. Silencing RUSC1-AS1 by siRNA significantly inhibited U2OS and HOS cell proliferation and invasion, measured by CCK-8 and transwell assay. Besides, knockdown of RUSC1-AS1 increased cell apoptosis in osteosarcoma cell lines. In addition, RUSC1-AS1 promoted the epithelial-mesenchymal transition (EMT) process of osteosarcoma cells. *In vivo* experiments confirmed that RUSC1-AS1 knockdown had an inhibitory effect on osteosarcoma tumor growth. Mechanically, we showed that RUSC1-AS1 directly binds to and inhibits miR-340-5p and activates the PI3K/AKT signaling pathway. In conclusion, our study demonstrated that RUSC1-AS1 promoted osteosarcoma development both *in vitro* and *in vivo* through sponging to miR-340-5p and activating the PI3K/AKT signaling pathway. Therefore, RUSC1-AS1 becomes a potential therapeutic target for osteosarcoma.

## INTRODUCTION

Osteosarcoma is the most common malignancy in bone, characterized by the presence of osteoid and immature bone tissues generated by malignant mesenchymal cells. Pulmonary and brain metastasis frequently occurs. Therefore, the natural course of osteosarcoma is dismal [1–3]. Osteosarcoma most frequently happens in adolescents, with the highest incidence in 15–19 years of age [4]. Current treatment for osteosarcoma includes preoperative induction chemotherapy, followed by definite surgery and postoperative chemotherapy. Although intensive treatment is given, the prognosis of osteosarcoma is still poor because of metastasis. The 5-year survival rate is less than 20% [5]. Elucidating the etiology and pathogenesis of osteosarcoma is necessary and new therapeutic targets are urgently needed.

LncRNAs are genome transcripts without protein-coding function, with a length of over 200 nucleotides. Although lncRNAs cannot translate into proteins, they can regulate protein expression. LncRNAs participate in various biological processes and play critical roles in disease progression, including cancers. One of the mechanisms by which lncRNAs function as oncogenes or tumor suppressors is to act as competing endogenous RNAs (ceRNAs) [6]. They can bind to miRNAs that regulate the expression of protein-coding genes, resulting in the overexpression of oncogenes. This regulatory mechanism has been widely studied in various kinds of cancers, including osteosarcoma. For example, lncRNA Taurine upregulated gene one can bind to miR-143-3p to regulate HIF-1α expression, thus promoting the metastasis of osteosarcoma [7]. LncRNA ODRUL promotes osteosarcoma development through miR-3182/MMP2 Axis [8].

LncRNA RUSC1-AS1 is reported to promote tumor growth in hepatocellular carcinoma [9], cervical cancer [10] and breast cancer [11]. Zhang et al. reported that RUSC1-AS1 is associated with unfavorable prognosis in osteosarcoma [12], but its role has not been studied yet. We confirmed considerable upregulated RUSC1-AS1 expression in the osteosarcoma patient samples and cell lines in the present study. RUSC1-AS1 knockdown significantly inhibited the proliferation and invasion, increased the apoptosis in osteosarcoma cell lines. *In vivo* experiments demonstrated that RUSC1-AS1 inhibition hindered osteosarcoma tumor growth. Furthermore, the direct binding of RUSC1-AS1 with miR-340-5p was confirmed mechanically.

## MATERIALS AND METHODS

### Cell lines and clinical samples

Osteosarcoma cell line MG63, U2OS, HOS and Saos-2, human osteoblast cell line (SV40 large T antigen transfected) hFOB1.19 were purchased from American Type Culture Collection (ATCC). Cells were cultured in DMEM-F12 medium (Gibco, USA) with 10% Fetal bovine serum (FBS) (Gibco, USA) supplemented with 100U/ml penicillin and 100 ug/ml streptomycin in 5% CO_2_ at 37°C.

Osteosarcoma tissues and adjacent normal tissues from 37 osteosarcoma patients were obtained from the department of orthopedics, Huazhong University of Science and Technology Union Shenzhen Hospital (2017.5–2020.8). Consent forms were signed by all participants. The diagnosis of osteosarcoma was based on pathological examination. Tissues used in the present study were put into the liquid nitrogen tank immediately after being removed from the body. Patients who had received any treatment before surgery, including previous surgery, chemotherapy, or radiation, were excluded from the study. This study was approved by the Ethics Committee of Huazhong University of Science and Technology Union Shenzhen Hospital.

### Cell transfection

Cell transfection was performed using Lipofectamine 2000 reagent (Invitrogen, USA). Briefly, 2 × 10^4^ cells/well were plated in a six-well plate the day before transfection. Transfection was performed according to the manufacturer’s instruction when the confluence reached 80%. si-RUSC1-AS1 was designed and synthesized by GenePharma (China). The target sequence of si-RUSC1-AS1 was 5′- ATGTTGGATAT CAAAGAGTATGA-3′. The sequence of si-NC was 5′-UUCUCCGAACGUGUCACGUTT-3′. For RUSC1-AS1 overexpression, the RUSC1-AS1 sequence was cloned into pcDNA3.1 (pcDNA3.1-RUSC1-AS1) and was synthesized by RiboBio (China). pcDNA3.1 negative control (pcDNA3.1-NC), miR-340-5p mimics (miR-mimics) and miR-mimics NC (mimics-NC) were purchased from RiboBio (China).

### Transwell invasion assay

Matrigel-precoated transwell chambers (8 um pore size, Corning, USA) were used for transwell invasion assay. In brief, 3 × 10^4^ cells were suspended in DMEM-F12 medium and were seeded into the upper chamber. The lower chamber was filled with DMEM-F12 containing 10% FBS. After 24 h incubation, cells invaded from the pores were fixed with 4% paraformaldehyde for 20 min and stained with 1% crystal violet. Invaded cells were counted using a microscope (200X, Olympus, Japan).

### Cell apoptosis assay

Cell apoptosis was measured through Annexin V+/Propidine iodide (PI)- flow cytometry assay. Briefly, 5 × 10^5^ cells were collected and stained with Annexin V and PI antibodies (BD Biosciences, USA) for 15min. BD FACSCelesta (BD Biosciences, USA) was used for cell apoptosis analysis. The results were analyzed by FlowJo software (FlowJo, version 9.3.2).

### Wound healing assay

2 × 10^4^ cells/well were seeded into 96-well plates the day before wound healing assay. The next day, when cell confluence reached 100%, wounds were generated using 10ul plastic pipette tips. Cells were cultured in DMEM-F12 medium supplemented with 1% FBS for 24 h. The results were analyzed using a microscope (100X, Olympus, Japan).

### Cell proliferation assay

Cell proliferation assay was conducted using Cell Counting Kit-8 (CCK-8, Dojindo, Japan). 3 × 10^4^ transfected cells/well were seeded into the 96-well plate and were incubated for 24, 48 and 72 h. 10 ul CCK-8 solution was added to each well at each time point and set the plate for 1–4 hours. The absorbance was read at 490 nm using a plate reader (Bio-Rad, USA).

### RT-qPCR

Total RNA was extracted from cells and tissues using TRIzol reagent (Invitrogen, USA). Reverse transcription was carried out using SuperScript III (Thermo, USA) according to the manufacturer’s instruction. Real-time PCR was conducted using SYBR Green (Thermo, USA) and was detected by Applied Biosystems 7500 Fast Real-Time PCR system. Primers used in the present study were listed in Supplementary Table 1. The relative expression of genes to GAPDH was calculated using the –2^△△Ct^ method.

### Western blot

Protein was extracted using RIPA buffer (Sigma-Aldrich). 25ug/well protein lysate was loaded onto an 8%–12% SDS-PAGE gel and was electro-transferred to a PVDF (polyvinylidene difluoride) membrane. The membrane was blocked in 5% non-fat milk for 30 min and was incubated with primary antibodies overnight at 4°C. The next day, we washed the membrane with TBST buffer and incubated the membrane with secondary antibodies. The membrane was developed by ChemiDoc MP System (Bio-Rad, USA). The antibodies used in the present study: p-AKT (Ser-473): #4060S, AKT (C67E7): #4691S, p-PI3K (p85/p55): #17366S, PI3K (p85): #4257S, β-ACTIN: #3700S were purchased from Cell Signaling Technology. Results were analyzed using ImageJ software (NIH, Version 1.53).

### Bioinformatic analysis and miRNA/lncRNA target prediction

KEGG pathway analysis was carried out using the KOBAS website tool (https://bio.tools/kobas). LncRNA-miRNA interaction was predicted by Starbase (https://web.archive.org/web/20110222111721/http://starbase.sysu.edu.cn/), ENCORI and miRDB (http://mirdb.org/) databases. Venn graph was depicted using Draw Venn Diagram Website Tool (http://bioinformatics.psb.ugent.be/webtools/Venn/).

### Luciferase reporter assay

Cells were seeded into a 12-well plate the day before transfection. After incubation for 24 h, cells were co-transfected with pmirGLO reporter vector containing RUSC1-AS1 MUT or WT gene and miR-mimics or mimics-NC. After transfection, cells were incubated for 48h. Luciferase activities were detected by the Dual-luciferase Reporter System (Promega, USA).

### RNA binding protein immunoprecipitation (RIP) assay

Magna RIP™ RNA-Binding Protein Immunoprecipitation Kit (Catlog.:17–700) was purchased from Millipore (US). RIP was conducted according to the manufacturer’s instructions. Briefly, protein lysate was incubated with magnetic beads at 4°C overnight. The next day, beads were washed with PBS buffer and RNA was purified using Proteinase K Buffer for RT-qPCR detection.

### *In vivo* experiment

2 × 10^6^ U2OS cells were injected into nude mice (BALB/c, 8-week-old female) subcutaneously. Mice were randomly divided into two groups and were treated with si-NC or si-RUSC1-AS1. For each mouse, 10nM si-NC or si-RUSC1-AS1 was dissolved in 100ul PBS and was injected into the tumor mass every three days. 28 days later, mice were euthanized, and tumors were fixed in 4% paraformaldehyde. Tumor volume was calculated using this formula: volume (mm^3^) = (length × width)^2^/2. The animal experiment was conducted following the recommendations in the Guide for the Care and Use of Laboratory Animals of the National Institute of Health. The research protocol was approved by the Committee on the Ethics of Animal Experiments of Huazhong University of Science and Technology.

### Statistical analysis

Results were shown as mean ± Standard Deviation (SD). The statistical analysis was conducted using SPSS software (IBM, Version 21.0.0). The paired Student *t*-test was used to compare RUSC1-AS1 expression in clinical samples. The independent Student *t*-test was used to compare the difference between the two groups. The one-way ANOVA test was used for comparisons in more than three groups. Kaplan-Meier Curve was used for survival analysis. The Pearson’s correlation coefficient was used for determining the relationship between RUSC1-AS1 and miR-340-5p. The Chi-square test was conducted to check the relationship between RUSC1-AS1 expression and clinical factors. All experiments were triplicated, and *P* < 0.05 was considered significant.

## RESULTS

### RUSC1-AS1 was highly expressed in osteosarcoma patient samples and cell lines

**Figure 1:**
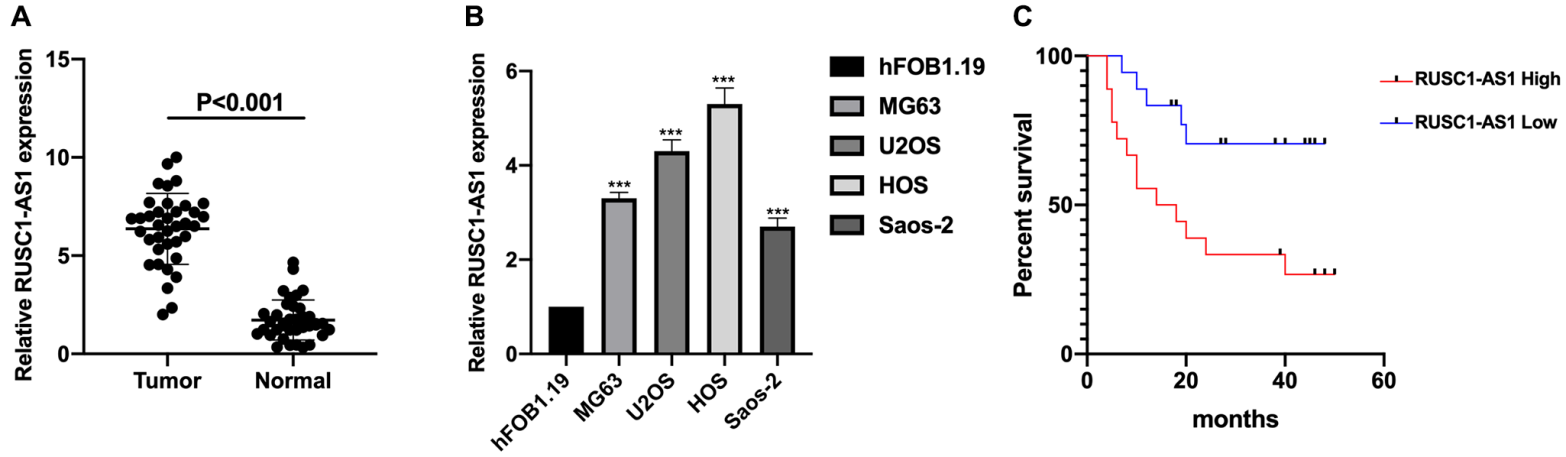
**RUSC1-AS1 was upregulated in osteosarcoma samples and cell lines.** (**A**) The expression of RUSC1-AS1 in osteosarcoma tissues and adjacent normal tissues. (**B**) The expression of RUSC1-AS1 in osteosarcoma cell lines MG63, U2OS, HOS and Saos-2 and osteoblast cell line hFOB1.19. (**C)** Kaplan-Meier curve of osteosarcoma patients with high/low RUSC1-AS1 expression. (^*^P < 0.05, ^**^P < 0.01, ^***^P < 0.001).

The expression of RUSC1-AS1 was investigated in osteosarcoma samples and cell lines firstly. As shown in Figure 1A–1B, the expression of RUSC1-AS1 was significantly upregulated in osteosarcoma samples and cell lines compared to that in normal controls and human osteoblast cell line hFOB1.19 (*P* < 0.001). We further examined the relationship between RUSC1-AS1 and clinical factors in osteosarcoma patients. The result showed that higher RUSC1-AS1 expression was associated with larger tumor size, advanced tumor stage and distant metastasis in osteosarcoma patients (Table 1). Further, survival analysis indicated that higher RUSC1-AS1 expression was related to shorter overall survival (OS) in osteosarcoma (Figure 1C). To further evaluate the prognostic value of RUSC1-AS1, Cox regression analysis was performed. The results showed that RUSC1-AS1 was an independent prognostic factor for osteosarcoma when considering clinical factors including age, sex, stage, tumor size, and metastasis (HR 2.541 (1.145–8.219), *P* = 0.022) (Table 2).

**Table 1:** The relationship between RUSC1-AS1 and clinical factors in osteosarcoma patients.

**Clinical Factors**	***N***	**LncRNA RUSC1-AS1**		***P* value**
		**High**	**Low**	
Age (y)				0.666
≥18	13	5	8	
<18	24	11	13	
Gender				0.065
Male	12	5	7	
Female	15	8	7	
Tumor Size (cm)				
≥5	21	17	4	0.002
<5	16	5	11	
Stage				
I-IIA	12	3	9	0.007
IIB-III	25	18	7	
Distant Metastasis				
No	13	3	10	0.005
Yes	24	18	6	

**Table 2:** RUSC1-AS1 was an independent prognostic factor for osteosarcoma.

**Variables**	**Patients (*N*)**	**Univariate Cox Analysis**		**Multivariate Cox Analysis**	
		**HR (95% CI)**	***P* value**	**HR (95% CI)**	***P* value**
**Age (y)**					
≥18/<18	13/24	0.892 (0.691–1.415)	0.495		
**Gender**					
Male/Female	22/15	0.971 (0.433–2.591)	0.882		
**Stage**					
I-IIA/IIB-III	12/25	2.688 (1.412–6.691)	0.042		
**Size (cm)**					
<5/≥5	16/21	1.981 (0.781–5.233)	0.069		
**Metastasis**					
No/Yes	13/24	2.335 (1.203–6.216)	0.002	1.892 (1.023–5.135)	0.013
**RUSC1-AS1**					
High/Low	18/19	2.913 (1.324–9.452)	0.001	2.541 (1.145–8.219)	0.022

### RUSC1-AS1 promoted cell viability, invasion and inhibited apoptosis in osteosarcoma cell lines

**Figure 2:**
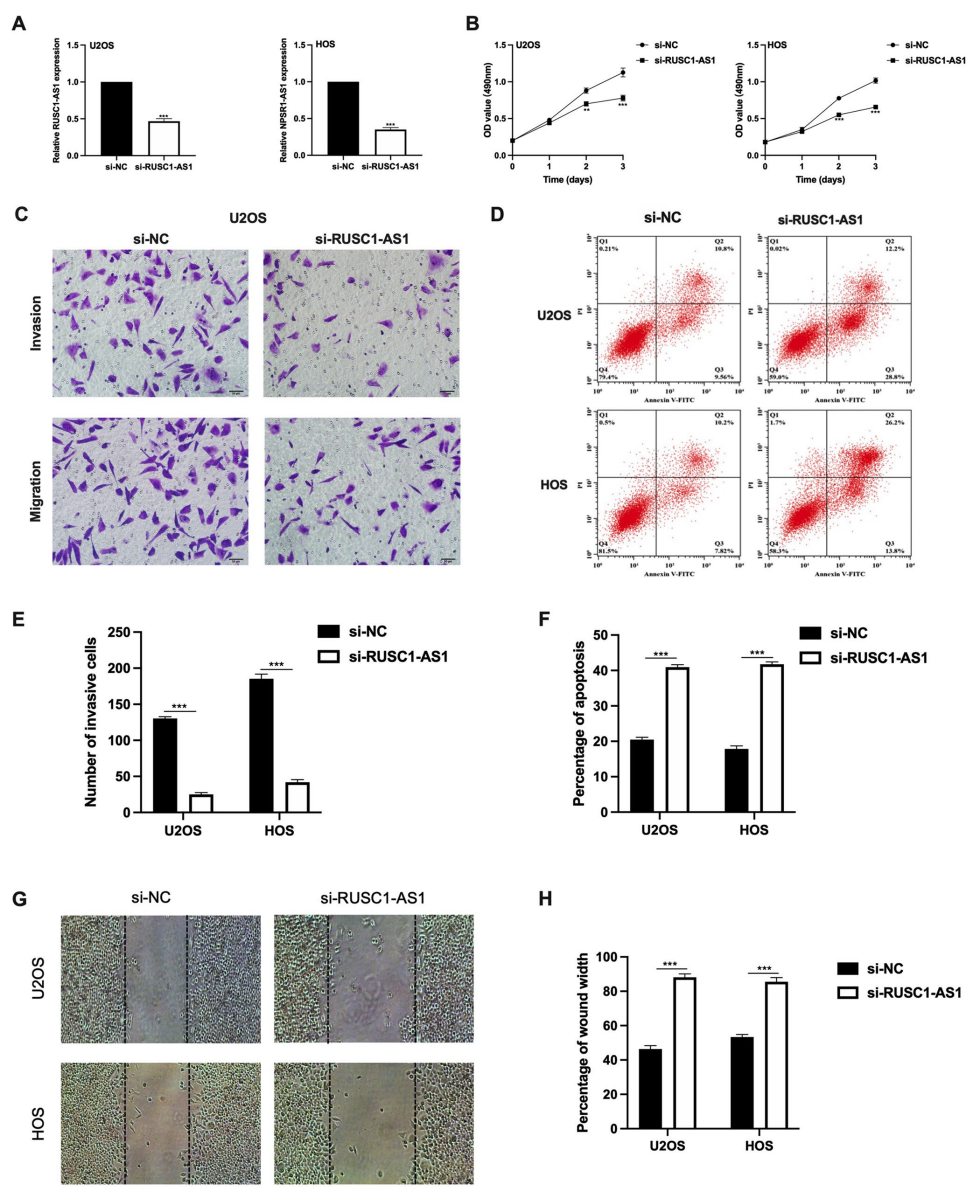
**RUSC1-AS1 promoted cell viability, invasion and inhibits cell apoptosis in osteosarcoma cell lines.** (**A**) RUSC1-AS1 expression after si-RUSC1-AS1 or si-NC transfection. (**B**) CCK-8 assay, (**C**, **E**) transwell invasion assay, (**D**, **F**) flow cytometry apoptosis assay, and (**G**, **H**) wound healing assay in U2OS and HOS cells after si-RUSC1-AS1 or si-NC transfection.

As RUSC1-AS1 was found upregulated in osteosarcoma cells, we further observed its function. SiRNA was transfected into U2OS and HOS cells for RUSC1-AS1 knockdown. The expression of RUSC1-AS1 was significantly lower after si-RUSC1-AS1 transfection in both U2OS and HOS cells confirmed by RT-qPCR (Figure 2A). After RUSC1-AS1 knockdown, we found that cell proliferation was significantly inhibited after 48 h transfection measured by CCK-8 assay (Figure 2B). In addition, the results of the transwell invasion assay and wound healing assay suggested that RUSC1-AS1 knockdown weakened the invasive ability of osteosarcoma cells (Figure 2C, 2E, 2G–2H). In contrast, after RUSC1-AS1 inhibition, cell apoptosis was significantly increased in the si-RUSC1-AS1 group compared to the si-NC group (Figure 2D, 2F).

### miR-340-5p was a direct target of RUSC1-AS1

**Figure 3:**
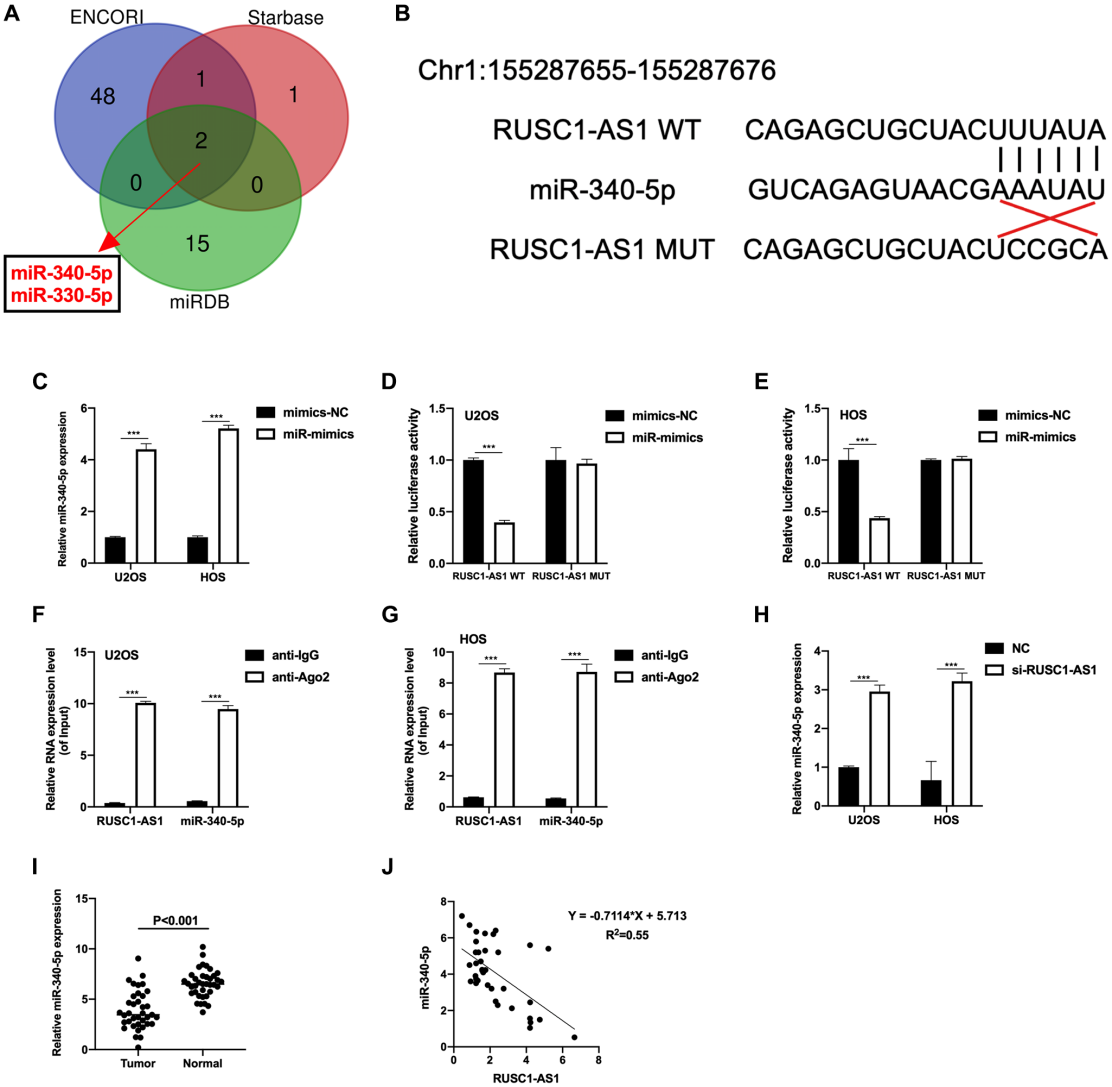
**miR-340-5p was a direct target of RUSC1-AS1.** (**A**) ENCORI, Starbase and miRDB predicted miRNAs that could bind to RUSC1-AS1. (**B**) Predicted binding sites of miR-340-5p and RUSC1-AS1. (**C**) The miR-340-5p expression after mimics-NC and miR-mimics transfection. (**D**–**E**) Luciferase reporter assay in U2OS and HOS cells showed direct binding of RUSC1-AS1 and miR-340-5p. (**F**–**G**) RIP assay in U2OS and HOS cells showed direct binding of RUSC1-AS1 and miR-340-5p. (**H**) The miR-340-5p expression after si-RUSC1-AS1 or si-NC transfection. (**I**) miR-340-5p expression in osteosarcoma tissues and adjacent normal tissues. (**J**) Linear correlation between miR-340-5p and RUSC1-AS1.

LncRNA often serves as a sponge of miRNA, thus regulates the expression of target genes [13]. Therefore, we used website tools (Starbase, ENCORI and miRDB) to predict and look for miRNAs that might bind to RUSC1-AS1. As shown in Figure 3A, miR-330-5p and miR-340-5p were the results of the intersection of three prediction tools, suggesting that these two miRNAs have a high possibility of binding with RUSC1-AS1. In subsequent experiments, we found that the expression of miR-340-5p was significantly decreased in osteosarcoma patient samples and cell lines, and it was inversely correlated with the expression of RUSC1-AS1 (Figure 3H–3J). Therefore, we speculated that there was a direct binding between miR-340-5p and RUSC1-AS1. To validate our hypothesis, luciferase reporter assay and RIP assay were conducted. The predicted binding site of RUSC1-AS1 and miR-340-5p was shown in Figure 3B. The transfection efficiency of miR-340-5p mimics (miR-mimics) was tested by RT-qPCR (Figure 3C). As shown in Figure 3D–3E, relative luciferase activity was significantly decreased in U2OS and HOS cells co-transfected with RUSC1-AS1 WT plasmid and miR-mimics, suggesting a direct binding of RUSC1-AS1 and miR-340-5p. Their binding was further validated by RIP assay (Figure 3F–3G). These results confirmed that miR-340-5p was a direct target of RUSC1-AS1.

### RUSC1-AS1 exerted its oncogenic role through miR-340-5p

**Figure 4:**
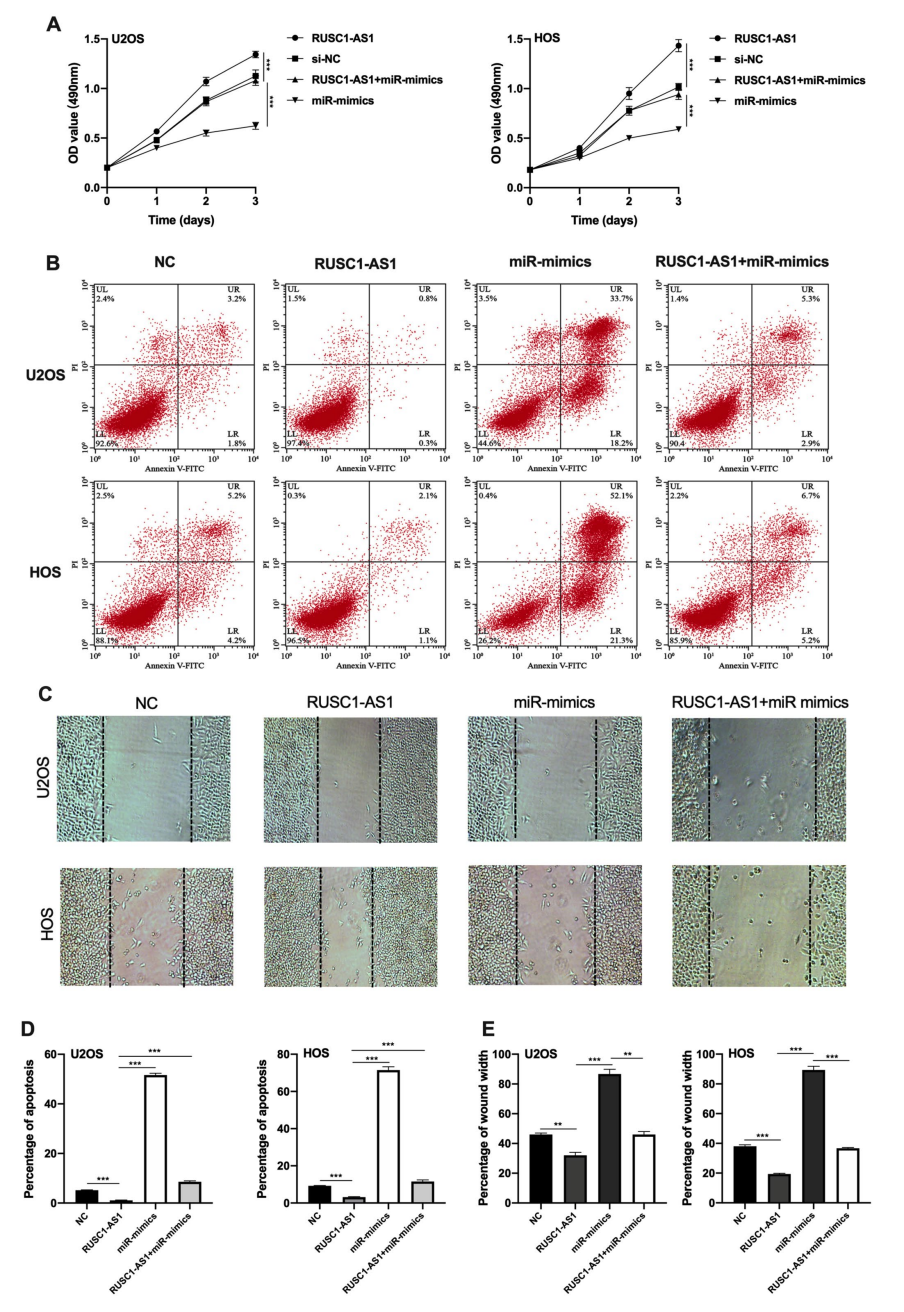
**RUSC1-AS1 exerted its role through miR-340-5p.** (**A**) CCK-8 proliferation assay, (**B**, **D**) flow cytometry apoptosis assay, (**C**, **E**) wound healing assay in U2OS/HOS cells or U2OS/HOS cells transfected with RUSC1-AS1 plasmid, miR-mimics or RUSC1-AS1+miR-mimics.

Next, we tried to explore whether RUSC1-AS1 promotes osteosarcoma progression through miR-340-5p. CCK-8 assay, flow cytometry apoptosis assay and wound healing assay were conducted using U2OS/HOS cells or U2OS/HOS cells transfected with RUSC1-AS1 plasmid, miR-mimics or RUSC1-AS1+miR-mimics. The results showed that the miR-mimics group had the least cell proliferation and the most cell apoptosis. The RUSC1-AS1 group showed the opposite result. There was no significant difference in the proliferation and apoptosis of osteosarcoma cells co-transfected with miR-mimics and RUSC1-AS1 plasmid compared with the control group (Figure 4A–4D). These results suggested that miR-mimics partially counteracted the oncogenic effects caused by RUSC1-AS1, implying that the RUSC1-AS1 exerts its oncogenic role through regulating miR-340-5p.

### RUSC1-AS1/miR-340-5p promoted osteosarcoma progression through the PI3K/AKT pathway

**Figure 5:**
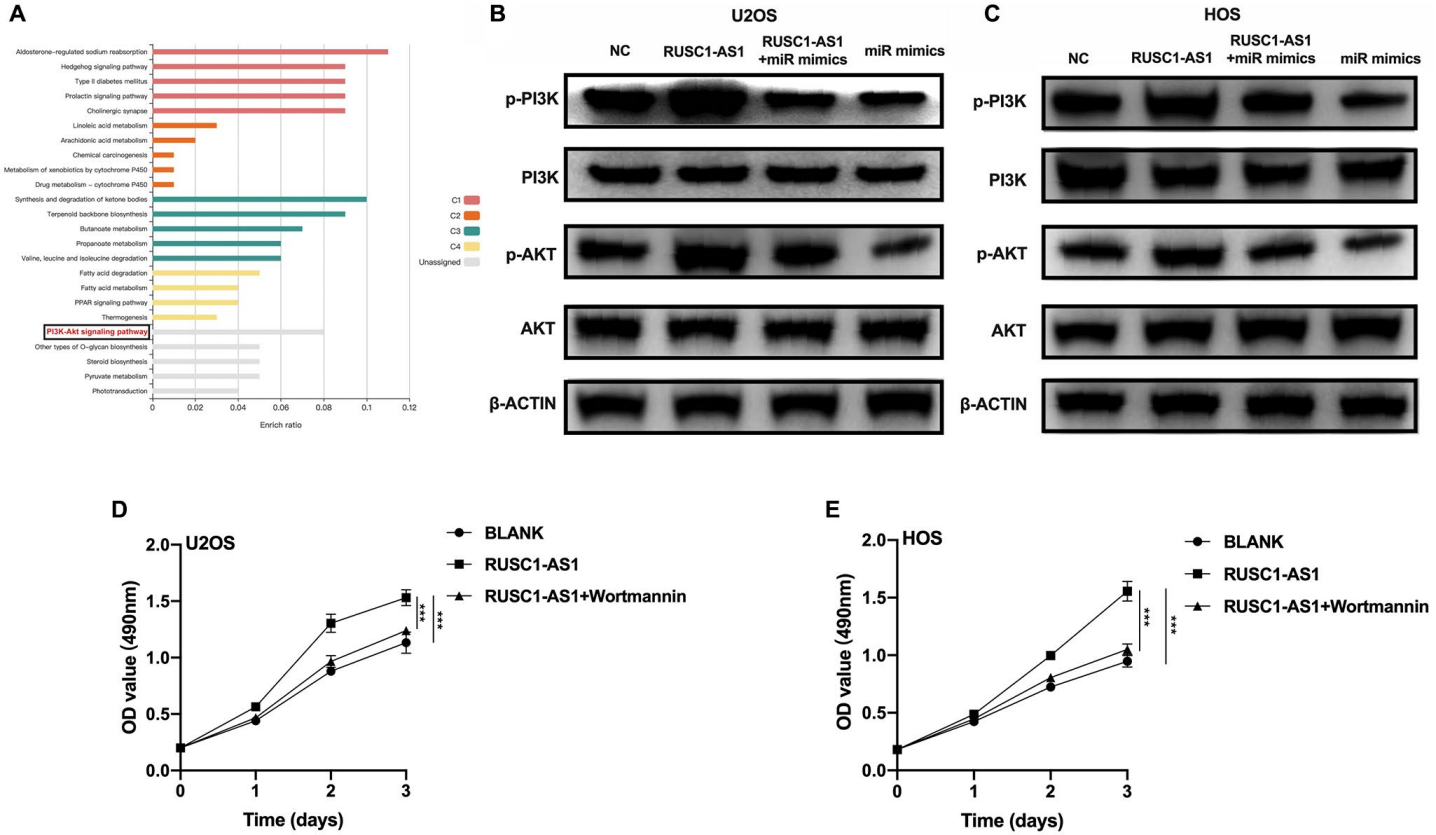
**RUSC1-AS1/miR-340-5p activated PI3K/AKT signaling pathway.** (**A**) KEGG analysis of the target genes of miR-340-5p predicted by miRDB website tool. (**B**–**C**) Western blot analysis of proteins in PI3K/AKT pathway in U2OS/HOS cells or U2OS/HOS cells transfected with RUSC1-AS1 plasmid, miR-mimics or RUSC1-AS1+miR-mimics. (**D**–**E**) CCK-8 assay in U2OS/HOS cells transfected with RUSC1-AS1 plasmid with/without wortmannin treatment.

To further explore the downstream signaling pathway of RUSC1-AS1/miR-340-5p, we used the miRDB website tool to predict the downstream target genes of miR-340-5p. We then performed KEGG enrichment analysis using these genes. The results showed that many target genes were enriched in the PI3K/AKT signaling pathway (Figure 5A). Therefore, we used U2OS/HOS cells and U2OS/HOS cells transfected with RUSC1-AS1 plasmid, miR-mimics, or RUSC1-AS1 +miR-mimics to perform Western Blot analysis. As shown in Figure 5B–5C, p-AKT and p-PI3K protein expression was significantly decreased in the miR-mimics group, while the opposite results were shown in the RUSC1-AS1 group. There was no significant difference in p-AKT and p-PI3K protein expression in the group co-transfected with miR-mimics and RUSC1-AS1 plasmid compared with the control group. To further verify that RUSC1-AS1 promoted tumor growth through the PI3K/AKT pathway, U2OS/HOS cells overexpressing RUSC1-AS1 were treated with the PI3K inhibitor wortmannin. The results showed that inhibition of the PI3K signaling pathway in RUSC1-AS1 overexpressed cells could effectively inhibit tumor cell proliferation induced by RUSC1-AS1 (Figure 5D, 5E). These results suggested that RUSC1-AS1 promotes osteosarcoma progression through binding with miR-340-5p to activate PI3K/ AKT signaling pathway.

### RUSC1-AS1/miR-340-5p promoted the EMT process in osteosarcoma cells

**Figure 6:**
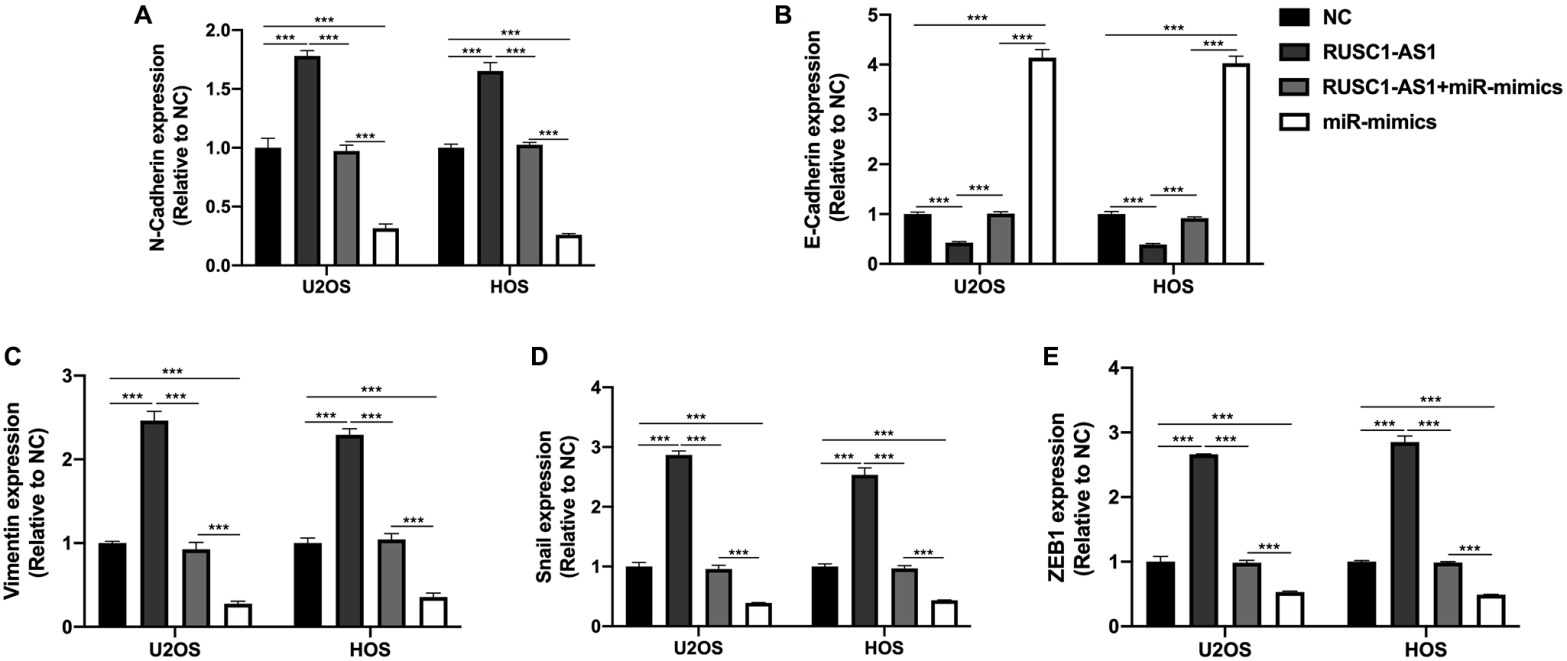
**RUSC1-AS1/miR-340-5p promoted EMT in osteosarcoma cells.** The expression of (**A**) N-Cadherin, (**B**) E-Cadherin, (**C**) Vimentin, (**D**) Snail and (**E**) ZEB1 in U2OS/HOS cells or U2OS/HOS cells transfected with RUSC1-AS1 plasmid, miR-mimics or RUSC1-AS1+miR-mimics.

EMT process was reported to be associated with the progression of osteosarcoma [14]. Therefore, we wanted to explore the effect of RUSC1-AS1/miR-340-5p on the EMT of osteosarcoma cells. We detected EMT-related genes N-cadherin, E-cadherin, Vimentin, Snail and ZEB1 in U2OS/HOS cells by RT-qPCR. As shown in Figure 6, we found that the expression of N-cadherin, Vimentin, Snail, and ZEB1 in RUSC1-AS1 transfected cells was significantly increased compared with the NC group. In contrast, E-cadherin expression was decreased considerably, suggesting that RUSC1-AS1 could promote EMT in osteosarcoma cells. The opposite results were seen in the cells transfected with miR-mimics. There was no significant difference in EMT-related protein expression between the RUSC1-AS1+miR-mimics group and the control group, indicating that RUSC1-AS1 acts as an oncogene by promoting EMT in osteosarcoma, and this process is partly through its direct binding with miR-340-5p.

### RUSC1-AS1 promoted osteosarcoma cell growth *in vivo*

**Figure 7:**
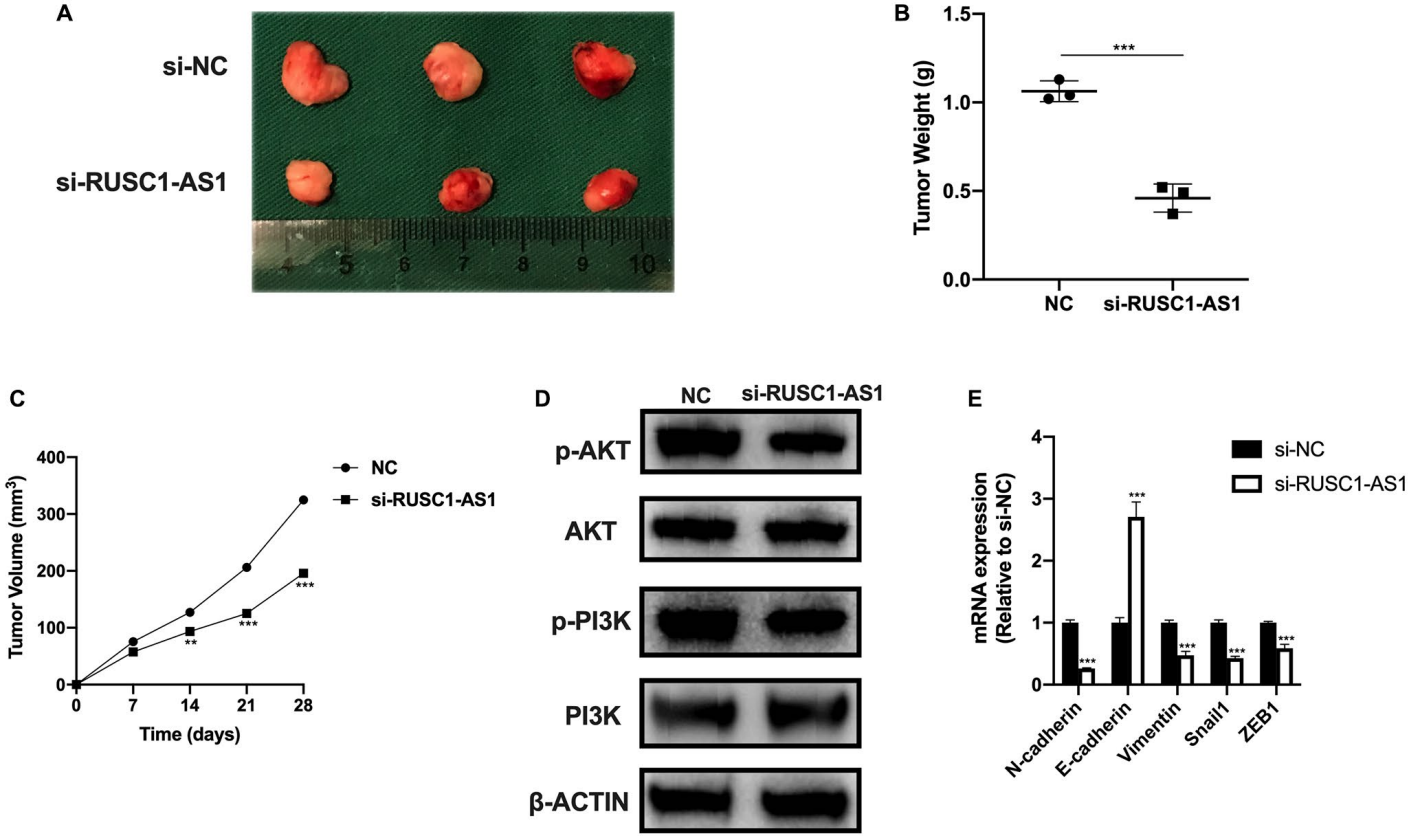
**RUSC1-AS1 promoted osteosarcoma cell growth *in vivo*.** (**A**) Tumor growth, (**B**) tumor weight and (**C**) tumor volume in U2OS injected nude mice treated with si-NC and si-RUSC1-AS1. (**D**) The protein expression of p-AKT, AKT, p-PI3K and PI3K in tumor cells from si-NC or si-RUSC1-AS1 treated mice. (**E**) The expression of EMT-related genes in mice treated with si-NC and si-RUSC1-AS1.

We also investigated the effect of RUSC1-AS1 on tumor growth *in vivo*. The results of *in vivo* experiment were consistent with those of *in vitro* experiment. Tumor growth was significantly reduced in the RUSC1-AS1 knockdown group compared with the si-NC group (Figure 7C). On day 28 of the *in vivo* experiment, we sacrificed the mice and found that the tumor volume and mass in the RUSC1-AS1 knockdown group were significantly decreased than those in the si-NC group (Figure 7A–7B). Western Blot analysis was used to detect the protein expression of the tumor cells. It was found that the PI3K/AKT signaling pathway was significantly inhibited in the si-RUSC1-AS1 group (Figure 7D). These results suggested that RUSC1-AS1 can promote tumor growth *in vivo*. In addition, we used qPCR to detect the mRNA expression of EMT-related genes. The results showed that compared with the si-NC group, the expressions of N-cadherin, Vimentin, Snail1, and ZEB1 in the si-RUSC1-AS1 group were significantly decreased. In contrast, the expression of E-cadherin was significantly increased (Figure 7E), suggesting that inhibition of RUSC1-AS1 could effectively inhibit the EMT process of tumor cells.

## DISCUSSION

Osteosarcoma is the most common primary bone malignancy. It is characterized by the uncontrolled proliferation of malignant mesenchymal cells and the formation of osteoid or immature bone tissue [15, 16]. It is most common in adolescents of 15–19 years. As osteosarcoma progression is rapid and the survival rate is extremely low, it becomes one of the life-threatening malignancies in adolescents [4]. As osteosarcoma is insensitive to radiotherapy [17], the main treatment methods are radical surgery [18] and polychemotherapy [19–23]. Despite recent advances in treatment, the survival rate for osteosarcoma today has not improved significantly compared with the 1980s [24]. Therefore, exploring the molecular mechanisms of the disease is crucial for understanding the disease progression and finding new treatment strategies.

LncRNAs are non-coding RNAs with more than 200 nucleotides. The most significant difference between lncRNAs and mRNAs is that lncRNAs are not protein-coding genes. However, they can positively or negatively regulate gene expression by regulating transcriptional/post-transcriptional or epigenetic steps. One of the most common ways of post-transcriptional regulation of lncRNA is to competitively bind with miRNAs and act as competitive endogenous RNAs (ceRNAs) to regulate the expression of target genes [25–27]. Recently, many studies have found that lncRNAs play essential roles in the occurrence and development of osteosarcoma. For example, 91H promotes the progression of osteosarcoma by regulating IGF2 expression [28–30]. BCAR4 promotes proliferation and migration of osteosarcoma cells by regulating the nonclassical Hedgehog/GLI2 signaling pathway [31–33]. HIF2Put promotes the growth of osteosarcoma cells by upregulating the expression of stem cell-related genes OCT4, SOX4 and CD44 [34]. Other lncRNAs can inhibit the growth of osteosarcoma. For example, MEG3 can induce the accumulation of p53 protein, promote the expression of relevant tumor suppressor genes, and inhibit osteosarcoma growth and development [35, 36]. In addition, some lncRNAs are closely related to the drug resistance of osteosarcoma cells [37, 38].

RUSC1-AS1 is reported to be a prognostic biomarker associated with unfavorable prognosis in osteosarcoma [12]. It is also reported to serve as a prognostic indicator of hepatocellular carcinoma (HCC) [9] and promote HCC proliferation through regulating the NOTCH signaling pathway [39]. Besides, RUSC1-AS1 serves as a ceRNA in cervical cancer by binding with miR-744 and thus increases the expression of the anti-apoptotic protein BCL-2 [10]. In laryngeal squamous cell carcinoma, RUSC1-AS1 is also reported to be a ceRNA as well as a prognostic indicator [40]. In breast cancer, RUSC1-AS1 plays its oncogenic role as an epigenetic regulator [11]. In the present study, we are the first to report that RUSC1-AS1 can promote proliferation, invasion and EMT of osteosarcoma cells and inhibit their apoptosis. Its function is achieved by directly binding with miR-340-5p and activating the PI3K/AKT signaling pathway. In addition, RUSC1-AS1 is associated with larger tumor size, advanced stages and distant metastasis in osteosarcoma patients. We also confirm that RUSC1-AS1 knockdown inhibits osteosarcoma progression *in vivo*.

The role of miR-340 as a tumor suppressor gene in osteosarcoma has been reported several times. It has been shown to regulate the Wnt/beta-catenin signaling pathway to inhibit osteosarcoma growth [41]. LncRNA OIP5-AS1 interacts with miR-340-5p to activate PI3K/AKT signaling pathway and inhibits the anti-tumor effect of miR-340-5p [42]. Also, miR-340-5p has been reported to enhance cisplatin-mediated apoptosis of osteosarcoma cells [43]. These results are consistent with our findings. Besides, miR-340-5p is also well studied in other human cancers. In thyroid cancer, miR-340-5p is upregulated and it promotes tumor cell proliferation through downregulating BMP4 [44]. In colon cancer, miR-340-5p acts as a tumor suppressor. Its inhibitory effect on colon cancer has been reported through different mechanisms. Algaber et al. reported that miR-340-5p inhibits colon cancer via targeting RhoA [45]. Yue et al. showed that NEDD4 triggers FOXA1 ubiquitination under miR-340-5p suppression [46]. Another study suggested that LINC00662 competitively binds to miR-340-5p to modulate the expression of CLDN8/IL22 in colon cancer [47]. These results all indicate that miR-340-5p plays an important role in the occurrence and development of human cancers.

In conclusion, our study demonstrated that RUSC1-AS1 promotes osteosarcoma progression and may serve as a therapeutic target for osteosarcoma.

## Supplementary Materials

Supplementary Table 1
