# Supplementary material for: LncRNA RUSC1-AS1 promotes osteosarcoma progression through regulating the miR-340-5p and PI3K/AKT pathway
Source: Aging (Albany NY). 2021 May 28;13(16):20116–30. doi: 10.18632/aging.203047 (PMC8436931; doi:10.18632/aging.203047)
Supplement: Supplementary Table 1 [file aging-13-203047-s001.pdf]

SUPPLEMENTARY TABLE

Supplementary Table 1. Primers for RT-qPCR.

PRIMER	FORWARD	REVERSE
RUSC1-AS1	TGCATTTGTTGTCCTGGATG	GCTGGTTTCAGGGTACAGGA
miR-340-5p	TCGGCAGGGUCAGAGUAACGA	CTCAACTGGTGTCGTGGA
N-cadherin	AAGGGAACATGAAAAGATAGTTAATTT	TGATGAAAAGGCTTCTGCTG
E-cadherin	GGGGGATTTGGTCAATGAT	ACTTTGAATCGGGTGTCGAG
Vimentin	GTTTCCCCTAAACCGCTAGG	GGAGAAGAGGCGAACGAG
Snail	CTCTAGGCCCTGGCTGCTAC	TGACATCTGAGTGGGTCTGG
ZEB1	AGGATGACCTGCCAACAGAC	GCTTCATCTGCCTGAGCTTC
GAPDH	AGCCACATCGCTCAGACAC	GTTAAAAGCAGCCCTGGTGA
